# Coping of Older Adults in Times of Covid-19: Considerations of Temporality Among Dutch Older Adults

**DOI:** 10.1093/geronb/gbab008

**Published:** 2021-01-10

**Authors:** Miriam Verhage, Lucia Thielman, Lieke de Kock, J Lindenberg

**Affiliations:** 1 Leyden Academy on Vitality and Ageing, Rijnsburgerweg AA, Leiden, the Netherlands; 2 Leiden University Medical Center, Department of Public Health and Primary Care, Albinusdreef AA, Leiden, The Netherlands; 3 Vrije Universiteit, Faculty of Behavioural and Movement Sciences, Educational Studies, Van der Boechorststraat 7, Amsterdam, The Netherlands

**Keywords:** Stress, Personal control, Qualitative methods, In-depth interviews, Crisis

## Abstract

**Objectives:**

Globally, mitigation measures during COVID-19 pandemic have focused on protecting older adults. Earlier disaster studies have shown the importance of including older peoples’ voices to prevent secondary stressors, yet these voices have received little attention during this pandemic. Here, we explore how Dutch older adults view this crisis and cope with measures to contribute to our understanding of coping of older adults in general and during disaster situations more specifically.

**Methods:**

Qualitative study using semi-structured telephone interviews with 59 diverse older adults aged 54 to 95 throughout the Netherlands.

**Results:**

Older adults typify this crisis as ungraspable, disrupting their daily and social lives. Despite filling their lives with activities, they experience loss or lack of purpose. They try to follow measures to decrease infection risk and gain control, and use problem- and emotion-focused coping strategies. Emotion-focused strategies used were interpreting their personal vulnerability, self-enhancing comparisons, acceptance, and distraction. In the latter two strategies, the temporary nature of measures was emphasized.

**Discussion:**

Older adults describe this crisis consistently with earlier findings from disaster studies. They use known coping strategies, but emphasize the duration in relation to their expectation of temporality. This underscores a dynamic, processual approach towards coping that incorporates temporal dimensions such as duration and order. Our findings stress the importance of acknowledging heterogeneity among older adults and adjusting communication about mitigation measures to decrease insecurity and increase resonance. This may make COVID-19 mitigation measures more manageable and age-responsible and allow older adults to start living again.

## Introduction

*It's elusive. […] It overcomes you. You don't know or aren't familiar with the cause. And you just have to wait and see (male, 77, married).*

Older adults suffer disproportionate impact during any disaster (Fernandez et al., 2002; Powell et al., 2009). Older individuals seem at higher risk of adverse outcomes of the disease during the COVID-19 pandemic. Globally, 94% of fatalities concern individuals above the age of 60 (Natale et al., 2020). Thus far, in The Netherlands, the country of interest in this article, a staggering 97.1% of those reported to have lost their lives due to COVID-19 are over the age of 60 (*Ontwikkeling COVID-19 in Grafieken 22-12-2020 [Development of COVID-19 in Charts]*, 22-12-2020). Mitigation measures have largely focused on protecting older populations through lockdown measures and social distancing (Bruinen de Bruin et al., 2020). Along with these measures come debates about isolating older adults (Armitage & Nellums, 2020; Becx, 2020; Gerrard, 2020), and an 'under-60' society (Nugent, 2020; van Gool, 2020). There may be detrimental effects of the enduring 'enforced loneliness' on health, in addition to heightened ageism and intergenerational divisions (Ayalon et al., 2020; Brooke & Jackson, 2020; Lichtenstein, 2020).

The overwhelming attention on older adults because of their physical risk is in stark contrast with the limited consideration of how they view and cope with this crisis. This is important as earlier disaster studies have shown that disregarding the voices and needs of older adults in crisis and post-crisis situations may cause additional risks and secondary stressors, such as anxiety, perceived loss of control and other mental health issues (Campbell, 2019; Ngo, 2014). Exploring how Dutch older adults experience and cope with the COVID-19 crisis, our overall objective is to highlight these voices, to shed light on their perspectives and needs and to help prevent these secondary stressors. Our main research question guiding this study is: how do Dutch older adults experience and cope with the COVID-19 crisis?

Moreover, this study aims to contribute to the understanding of coping during disaster situations and, in a more limited way, to coping processes of older adults more generally. Studies exploring coping during the aging process and disasters more specifically, are inconclusive on how older adults cope. Some studies suggest that older adults cope better because of life experience and lower psychological vulnerability (Barusch, 2011; Ngo, 2001; Whitbourne, 2005) and studies during the first weeks of COVID-19 seem to confirm this showing lower reactivity to stressors (Klaiber et al., 2020). This is in line with core gerontological theories on successful aging demonstrating that they are able to adapt, select and optimize in seemingly adverse conditions (Albrecht & Devlieger, 1999; Baltes & Baltes, 1990). Conversely, some scholars argue that older adults are less able to cope with sudden crises because they are less open to change, new events and uncertainty (Donnellan & Lucas, 2008; Roberts et al., 2006). These contradictory findings may be due to the conceptual lenses these studies have taken; the latter sees coping more as a personality disposition, while the first sees coping as a process in context, consistent with the transactional theory of coping of Lazarus (TTSC, Folkman & Moskowitz, 2004; Lazarus, 1993), which sees stress as a transaction between an individual and their environment and coping as a process based in cognitive appraisals and behavioral responses.

With this qualitative research into coping of older adults during the COVID-19 crisis, we aim to contribute to the relatively limited body of work on coping of older adults in disaster situations (some exceptions are Adams et al., 2011; Campbell, 2019; Henderson et al., 2010; Liddell & Ferreira, 2019) with an empirical examination of coping during the crisis itself in which situational and contextual factors are taken into account through a qualitative approach. We draw upon original data from 59 semi-structured telephone interviews with older adults with diverse backgrounds throughout the Netherlands (see Table 1.) We describe their views and relate these to the existing literature on coping during the aging process and disasters. On the basis of our findings, we reflect upon implications for the study of coping and the COVID-19 mitigation measures in The Netherlands.

## Methods

### Ethical approval

This study was approved by the Institutional Review Board of the (BLINDED FOR REVIEW) for observational studies, registered under number CoCo 2020-014.

### Data collection

We conducted 59 semi-structured telephone interviews using a topic guide (see Supplementary Material) between 27 March and 20 April 2020. Although telephone interviews have been shown to provide less detail, using specific interview techniques (e.g. responsiveness) can enhance this (Irvine, 2011; Irvine et al., 2013). Moreover, telephone interviews can increase feelings of anonymity making respondents more relaxed and open, decreasing interviewer effects (Michiniello 2008, Sturges & Hanrahan 2004). Given the empirical questions and regulations due to lockdown measures in the Netherlands, we felt telephone interviews to be a suitable method of data collection. The average duration of the telephone interviews was 45 minutes (range 27 – 80 minutes). We interviewed 63 individuals, but we excluded the data of four interviewees because we felt uncertain of whether they fully understood the purpose and possible consequences of participating.

### Sampling

Given the lockdown measures, we reached out to potential respondents using snowball sampling using personal networks and their networks of networks to diversify the sample (Bernard, 2011). This approach of starting from personal relations would likely result in higher response rate (Kirchher & Charles, 2018). Following our explorative question, we aimed to ensure diverse perspectives by taking into account relevant background factors: gender, age, living situation, residence, and socio-economic situation. To enhance sample coverage, we used several sources (i.e. persons) for snowball sampling (respondent-driven sampling). We aimed to include individuals aged 60 and above. We chose this relatively young threshold because it is known that individuals in more precarious living situations experience age-related challenges on average at a chronologically younger age than others. During the interviews we interviewed one individual living in a long-term care facility suffering from cognitive decline who was 54 years old. Given that we aimed to include individuals experiencing age-related problems drawing upon the geriatric syndromes (Inouye et al., 2007), we decided to not exclude this individual. After including approximately 40 interviewees, we did not hear novel themes and reached data saturation, then cross-checked our sample for diversity (Bernard, 2011). To ensure heterogeneity of our sample on the aforementioned characteristics, we then continued with purposeful sampling, to ensure our depth of understanding included the anomalous cases. We completed data collection on 20 April 2020 (see Table 1.).

[insert Table 1. about here]

**Interviews**

We used an interview guide that included neutrally formulated themes specific for COVID-19 (e.g. COVID-19 measures, experiences during the crisis, and dealing with the crisis). We added themes based on existing knowledge of domains of importance for aging well among older adults (Gabriel & Bowling, 2004; Steptoe et al., 2015). We had open discussions with two older adults about the topic list the week of 20 March 2020. This resulted in the final topic guide (see Supplementary material). Each theme (e.g. daily life, social life) had a number of hints for possible open questions. Given the explorative nature, our interest in subjective experiences and the interpretative approach underlying our study, interviewers were free to add topics based on respondents' answers and formulate their own open questions during the interviews guided by the interviewee's experiences.

**Procedure**

Potential interviewees were contacted by a personal contact to ask whether they could be approached for participation. A researcher (BLINDED FOR REVIEW) then informed them about the study, its purpose, procedures, and possible consequences. Interviewers tried to make sure that there were several days between this first contact and the actual interview to give sufficient time to consider participation, but some explicitly declined this. Before the actual interview, respondents were again asked for informed consent and the actual consent was, after permission, repeated and audio-recorded. Three interviews, with residents of long-term care facilities, spanned several weeks upon participants' requests due to fatigue, inconvenient timing or because they enjoyed the conversation. Interviewees indicated when they wanted to stop and interviewers called them again at their convenience to continue the interview. Each time the interviewer repeated informed consent and would briefly summarize what had been said to continue to topics not yet covered. All interviews were, after permission, audio recorded and then transcribed verbatim.

During data collection, the authors had weekly team videocalls to reflect upon their possible influence on the data. We noticed that although interviewer response effects were smaller than in face-to-face interviews, we used more prompts during the interviews. Since body language was absent, we needed to translate visual cues into verbal cues, resulting in more confirmative prompts such as 'yes', 'indeed', and 'I see'. Similarly, we used more confirmative cues when showing empathy, resulting in the interviewer spending more time talking to relate their understanding. Overall, it seemed that during the telephone interviews the interviewers spent more time talking than in face-to-face interviews.

**Analysis**

Considering the explorative nature of our study and our aim to follow the subjective experiences of older adults and their interpretations, the authors used open coding, leading to the identification of themes and patterns using a computer assisted qualitative data software MAXQDA (release 2018.2.4) in a password secured project. Several themes emerged which were then compared resulting in two main themes central to this article: perceptions of the crisis and coping with the crisis. Documents (transcripts) received unidentifiable codes consisting of numbers and letters. We then used multiple coding and compared the codes of different researchers to refine our coding framework (1st tier triangulation). After coding all interviews, we started constant comparisons (Glaser & Strauss, 1967) between the interviews and compared the codes to discover relations between them and discussing alternative interpretations. In this phase, the authors met weekly to identify themes and patterns, first focusing on descriptive findings before continuing to the how and why of these subjective experiences of older adults. All interview fragments presented in this article were translated by the authors. These citations are accompanied by demographic details and living situation. Unless otherwise stated, cited interviewees lived independently in their own homes.

## Results

In this article, we first delve into older adults' views on the COVID-19 crisis to describe how they situate this crisis to highlight their voices and to provide the context in which coping strategies were employed. We then present several strategies that older adults used to cope with the crisis.

### Situating the crisis: Meaning in life

*Because it is so… ungraspable. If I cycle and I come across a lion, I know this is dangerous. And this is even more dangerous, just intangible. You can't see it (male, 67, married).*

Disasters are characterized by their disruptive nature (Campbell, 2019) and older adults typify the COVID-19 crisis no differently. The sudden changes in their everyday lives were described as 'surreal' and 'unbelievable'. This was further underscored by their perception of the virus as invisible and hard to understand in terms of contagion, risk, and chances of survival. Many described how they felt being right in the middle of the crisis, while experiencing the virus as something totally beyond oneself and one's control.

Having to put their individual lives on hold was designated as having the largest impact on most of the interviewees' daily lives. Interviewees described how they were suddenly confronted with holding off ambitions and plans they had, which varied from celebrating a birthday, writing a book to travelling. Some older adults described this loss of purpose in life as "depressing" (female, 73, living alone) and "anxious" (female, 65, living alone).

Seemingly minor activities such as getting groceries, shopping or doing sports were described by some as very meaningful, perhaps because these were things that were now done by others or no longer (easily) accessible. But also because doing these seemingly simple things gave them structure, a sense of self-determination and freedom:

*If they [home care and hairdresser] visit you, it breaks your day and you have a day structure [...] The only thing is, I used to go to the swimming pool every week. Well that is not possible now. And well, swimming in the water is pretty lovely when you walk with a walker and you're in the swimming pool and you can walk again (female, 92, living alone).*

Older adults felt a loss of meaningful activities and described how they were waiting for 'real' life to start again so they could engage in valuable activities.

On top of this, as for other age groups (Backer et al., 2020; de Klerk et al., 2020), the social lives of older adults have been limited and changed drastically since the COVID-19 mitigation measures. For most, this further contributed to their feelings of loss of meaning. They explained how social contact made them feel "valued", "seen" (female, 73, living alone) and "meaningful" (female, 74, living alone). Engagements in volunteer work, informal care, babysitting, leisure clubs or for a few self-employment were halted. Especially those living alone or those who had become a fulltime caregiver now mentioned that they missed others so that they could discuss, reflect and break their ruminations, which "widened their world" (female, 77, living alone):

*Who sits with me at my table and has a cup of soup with me? No-one. That is a kind of loneliness, of being alone. It makes it more difficult to make deliberations for yourself, because no-one replies (female, 74, living alone).*

A related but differently experienced theme was the lack of meaning in life some interviewees faced already before the crisis began. A few felt their feeling of not really mattering momentarily lifted at the beginning of the crisis. They disclosed how they enjoyed the additional attention they now received as 'vulnerable' older adults: "Before, I was no-one, and, well, now I am someone. I am phoned and I receive cards and flowers" (female, 92, living alone). This attention made them feel acknowledged and appreciated, like they mattered to others:

*In general, it's like long-term care staff just do their job and you are kind of by chance the person taken care off and when they're done, they have done their duty and close the door. But I find it extraordinary that in these times they manage to have attention for you. It makes you feel like you exist (male, 70, married, living in long-term care facility).*

A few weeks later, however, most of the residents of long-term care facilities, whom we interviewed in intervals, started to feel differently. They were not allowed to leave their rooms, and visitors were not permitted. Staff members were generally very busy managing COVID-19 infections among inhabitants and there was a shortage of staff as a result of infections among them. While earlier their lack of meaning was eased, it was now strongly emphasized in social isolation, as the male cited above reflected after three weeks: “they probably haven’t forgotten me, but at the moment it gets to me. Though you really need that [attention] now, you are disconnected”. Interviewed older adults thus appraised the COVID-19 crisis as largely beyond their control. After a few weeks many described experiencing a loss or lack of meaning in life, yet they found their own ways to cope with the situation.

## Coping strategies during the crisis

We next delve into the patterns of the described coping strategies. There are many ways to categorize coping strategies (Skinner et al., 2003), but most commonly they are grouped in three main classes: problem-focused, emotion-focused and meaning-focused coping. The first strategy tries to cope with the stressor by resolving the problem if it is controllable. The second changes the way you relate to the stressor, while the third focuses on finding meaning in the stressor (Blum et al., 2012; Carver, 2013). Meaning-focused coping is sometimes categorized under emotion-focused coping. Moreover, the used strategies can in practice fall under several classes. Given the experienced ungraspable nature of the crisis, interviewees largely resorted to emotion-focused and meaning-focused coping strategies, and less prominently included problem-focused strategies. We outline these in more detail below.

### *Self-enhancing comparisons*

One of the most important emotion-focused strategies, irrespective of the interviewees’ personal circumstances, was self-enhancing comparisons. Depending on their personal situation they pointed to those living alone, with little income, living in long-term care facilities, families with children or homeless persons:

*There are so many people in worse circumstances… Such as people living alone, homeless people, people in asylum, you name it. People in bigger cities, in small apartments, in long-term care facilities not being able to receive visitors (female, 82, married).*

These kinds of comparisons allowed them to maintain their self-esteem and self-confidence. Many interviewees situated this within a context of vulnerability, in which others' situations were deemed beyond one's control to a greater extent than their personal situation. Self-enhancing comparisons allowed older individuals to lessen the emotions they experienced, increasing their sense of control and self-image.

***Gaining control by following measures***

At the time of the interviews in the Netherlands the most important mitigation measures were hand hygiene, staying home as much as possible and avoiding social, face-to-face contact (see Bendien & Abma, 2020). In general, interviewees tried to implement the imposed measures according to their interpretations in the context of their personal situations to reduce the risk of contracting the virus, a problem-focused strategy:

*I am not going to think 'I want this and I want that'. That is fooling yourself of course. Be realistic and follow the rules. And live by the rules you always had, your own rules I mean, then there is still something of your own [laughs] (male, 79, living together).*

Following their own interpretations of measures was by some described as an act of solidarity: "If I buy groceries, I wear latex gloves and I bring my own shopping trolley so the girls don't have to clean it […] simple, daily things by which you indicate that you also think about others" (female, 72, living alone). For others this strategy was not just a problem-focused strategy, but also an emotion-focused strategy to decrease insecurity and regain control in a surreal and ungraspable situation:

*I am also scared. I'm not just going out, I'm very careful when I'm outside. After I buy groceries I immediately wash my hands with soap. Then I leave the groceries for a bit in the hallway and then I clean everything with a wet cloth before I put it in the cupboards. (female, 65, living alone).*

This more emotion-focused strategy seemed to be described more often by interviewees with language difficulties and those with less resources, especially social capital (i.e. an individual's ability to have, access and utilize social connections influenced by the structural access to and ability to exert power) (Bourdieu, 1986). They had limited opportunities to share daily chores or discuss and share worries with others and this was something they *could* do. However, for some this strategy did not work and trying to interpret measures left them feeling insecure:

*I don't have a piece of bread in the house, but I don't dare to go out, because I am scared of dying and I live in fear to go out, because I might catch something (female, 79, living alone).*

***Distraction***

Another emotion-focused coping strategy described by interviewees was trying to distract themselves and focus on other things. The distraction kept time moving and allowed them to have time for things they would not normally do. They found meaning in these distractions, which allowed them to cherish small things and clear their minds by engaging in (new) leisure activities. It also prevented them from worrying or feeling powerless and created a buzz around them, especially for those living alone:

*And now, when I arrive home, if you don't take your phone, there is silence. Then it is me and the television and then I watch all these strange programs or I go watch repeats and I try to have sound around me. […] Not that I am dissatisfied, after all I am healthy, but it does make me sad. (female, 63, living alone).*

Distraction was also a way of coping with social and societal disengagement. Such distraction was not typified as useless, but as less meaningful than activities before the COVID-19 mitigation measures in the Netherlands:

*Everything is… I can't sing, I can't go to the care home. Everything is… those are the things that are very nice. But well, I can entertain myself. I have a lot of nice contact with friends, over the phone. I get through the day. (female, 80, living together).*

***(Temporary) acceptance***

One other emotion and meaning-focused coping strategy described by many interviewees was simply to accept the change and to "live with it" (male, 95, living alone) for the time being, which would allow them to enjoy even more after the pandemic:

*This you also have to accept. You have to wait until everything is free again. Then you can enjoy double. If you have gone through this, in the beginning you will look at things differently. You will be happy that things happen, that you can go out, pay a visit. For now we have to just bear through[.] (female, 92, living alone).*

However, acceptance and following the measures was for some not unconditional; they emphasized that their expectations that the measures were temporary made it possible to adhere to these strategies. They explained that they simply had to keep going before they could start living again: "In a few months this will pass. […] But emotionally, now, I find it kind of boring" (male, 72, living together). Such temporal scopes were described as providing a sense of security and control. Yet, some older adults were quite conscious of the uncertainty inherent in this temporary nature. They approached this insecurity by not thinking too far ahead to avoid disappointment:

*Not planning for the long term. You can think about what you do today, but really looking into the future you can't do that now. […] that's a shame. I don't get depressed, but I find it a a shame. I mean, you start to think about things. (male, 67, married).*

***Interpreting individual 'vulnerability'***

An emotion-focused coping strategy more specific to the COVID-19 crisis was dealing with the flow of information to interpret individual vulnerability, but also to reduce fear. For some, this meant following the news very carefully. One particular case was an older adult infected with COVID-19, who did so to find hope and reassurance: "a mini-research about mice or rabbits, infected but none of them got it again. Those small things, you are craving for news that is in your advantage, which can decrease your fear" (male, 68, married). Others did quite the opposite and filtered or selected the news to worry less: "It only becomes bigger in your head" (female, 73, married).

Moreover, the filtering of the news had another motivation. Some older adults described that the representation of older adults as vulnerable throughout the media reduced their sense of control and made them feel incompetent: "At first I was so outraged. When you are older, you are thus vulnerable. No, older people and vulnerable older people that is something else" (female, 73, married). They felt that among older people there are many differences in abilities and age alone does not equal vulnerable; thus, they made their own estimations of what it meant to be vulnerable. Numerous interviewees stated in various ways "what is meant by vulnerable? Is that just physically vulnerable or mentally vulnerable? There are so many gradients [..] I don't feel addressed" (male, 72, married). The conjoining of vulnerable and older was by some experienced as disregarding the heterogeneity among older adults and made some feel as if the competence of older adults was thoughtlessly doubted:

*Just like older adults are a bit disabled, while there are pretty modern older adults that can videocall and all that. Actually, we should use the strength of older adults and not just put them in a corner like 'oh you are vulnerable and pathetic, so stay inside' (female, 63, living alone).*

The COVID-19 virus was thus a sudden confrontation with one's own vulnerability and engendered among most participants paradoxical feelings due to belonging to but not identifying with a vulnerable group:

*Well, I find it a good thing that they represent older people as a vulnerable group. I think I am part of that group with 67 years. I am not sure, but… in my own perception I am not at all 67. I don't feel I belong to this group. I just have to be careful (male, 67, married).*

But opinions were diverse, which was reflected in their reactions to initiatives for older 'vulnerable' people, for instance to a special 'senior hour' in supermarkets:

*Oh no, I just do my groceries. […] I mean I know I am old, or at least older, but I do not go to those senior things of shops. No, not for me. What they [other age groups] do to older adults to enjoy themselves (female, 73, living alone).*

*I find that they do take older adults into account, I find that extraordinary that everyone is stimulated to wave extra, to drop by at a distance, or send a postcard, flowers, yes I find that excellent (female, 92, living alone).*

The equation of old with vulnerable was by some interpreted as not applicable to them and made them feel at lower risk than 'real older adults'. Others assessed their individual risks to justify which mitigation measures they would follow and which they would not (i.e. going out, getting groceries or visiting relatives).

**Discussion**

By exploring the voices of older adults living in lockdown as a result of COVID-19, our overall objective, we have demonstrated that they have put their lives on hold and emphasize the importance of purpose in life. This fits with findings in other disaster situations (Shaw et al., 2014) and lines up with gerontological research (Ryff & Singer, 1998) that shows positive associations of purpose in life with mental and physical health and wellbeing (Pinquart, 2002).

We found that older adults experience this crisis in diverse ways and their resources vary; especially important in this crisis is their social capital (Bourdieu, 1989). In the Netherlands, those living alone and those in long-term care facilities struggled because they were not sharing their households and access to other social capital was simply cutoff.

Interviewees use various known coping strategies to deal with this crisis, such as self-enhancing comparisons (Fry & Keyes, 2010) and acceptance (Meyer, 2017). They interpreted and implemented lockdown measures according to their own situations and perceptions, distracted themselves and focused on other things. The coping strategies found fit with the TTSC and a more processual, situational approach towards coping acknowledging coping as a complex interplay between individual distinctions, contextual factors and characteristics of the event itself, requiring a more detailed-oriented approach (Blum et al., 2012; Carver, 2013; Greve & Staudinger, 2015). Strategies employed can largely be categorized as emotion-focused, which helped participants regain control and increase self-determination and feelings of competence. Following mitigation measures to reduce the risk of infection, however, seemed to be both a problem and emotion-focused strategy.

Many interviewed older adults had difficulty with the equation of older people with vulnerable people (cf. Furedi, 2007) and their individual interpretations of 'vulnerability' framed their personal estimations of risk and decisions on mitigation measures. This appraisal of vulnerability and the at times explicit rejection of 'vulnerable older adults' may be a way of reducing perceived loss of control found in earlier disaster studies (Campbell, 2019).

In several of these coping strategies, interviewed older adults emphasized the temporary nature, both the duration as well as the experienced clarity and unclarity around this, of the mitigation measures. This may be related to the strategies being overwhelmingly emotion-focused as little could be done about the occurrence of the virus and few found meaning in this crisis. The adherence to temporality may also be a way to decrease insecurity, as earlier post-disaster research has shown that long-term insecurity may be even more detrimental than the event itself (Shaw et al., 2014). This seems specific to the COVID-19 crisis, yet clarity on the temporal dimension of adversity may not only be of importance to coping during disasters, but also of influence in processes of adaptation and coping during the aging process. Despite recent attention to the influence of coping skills before the stressor occurs (Neupert et al., 2019), other temporal dimensions have rarely been taken into account explicitly. With regards to coping more generally, this raises questions about the duration and changes in the process of coping: When and how does the process shift from acceptance to rejection, for instance? And how is timespan relative to expectations of adversity of influence?

In this view, coping is not just an interaction between person and context, but a dynamic process in which temporal dimensions such as duration, timing and order of stressors may play an important role (cf. Carver, 2013). A longitudinal or recurrent qualitative approach, as argued by others (Nevedal et al., 2019), may highlight dynamics within the process of coping in which expectations related to the duration of the stressor can be taken into account. Drawing on existing evidence (Coleman et al., 1999, 2007; Richardson et al., 2014), this may enhance our understanding of adaptation and coping also in the process of aging, which has largely been focused on the outcomes of coping (such as wellbeing and mental and physical health) and less on how the process unfolds.

### *Strengths and limitations*

Two strengths of our study are the heterogeneity of our sample and the large number of interviews, which thereby capture an in-depth view of the diverse patterns of interpretations among older adults in the Netherlands.

One possible limitation is the use of telephone interviews, which may have resulted in less depth than face-to-face interviews. However, telephone interviews may also reduce interviewer effects and create feelings of anonymity supporting disclosure and openness.

We only used one method in this study and triangulation would have led to even richer findings, as well as recurrent interviewing. Given the COVID-19 restrictions, and our desire to include individuals with low literacy and language difficulties, we felt our qualitative approach was most appropriate. Moreover, this study can contribute to the development of more quantitative approaches by identifying coping strategies and temporal considerations. Quantitative studies can research relations between coping strategies at different time points, significant patterns in coping strategies identified and the impact of temporal considerations on, for instance, experienced stress and anxiety.

One key limitation is that the Netherlands is one specific country and findings may be different for other countries. Although mitigation measures have been similar across the world, views and interpretations of older adults may differ.

### *Conclusions and Implications*

In this article, we have shown that older adults view the COVID-19 crisis as disruptive and feel that their lives have lost or lack meaning. We also found that some older adults seem to struggle more than others, such as those living alone. It is therefore of utmost important to find ways to find meaning in life, including social and societal engagement, accessible to older adults, yet with clear and unequivocal measures to reduce risk of infection, fear and insecurity about personal vulnerability. We also need to recognize and involve older adults as experienced experts, as this can help find ways to make COVID-19 measures manageable and age-responsible, and allow older adults to start living again. We found personal estimations, insecurities and anxiety among older adults who barely and ambivalently identified with the predominant designation of vulnerable. This points to the necessity of clear and unambiguous communication about mitigation measures to reduce insecurity and fear. It also highlights the necessity to take into account processes of identification with terms such as ‘older people’ and ‘vulnerable’. In communicating measures, we need to use language that fits with the lifeworlds of older adults and avoids ageist connotations.

Older adults use various coping strategies to deal with this crisis that draw our attention to the dynamic, changeable and temporal dimensions in the process of coping, especially with regards to the expectations of duration of stressors as a way to enable coping. Our study may be a first stepping stone for further quantitative, qualitative and longitudinal coping research during disasters and the ageing process. This empirical investigation shows that holding off living, acceptance and distraction are acceptable in the short-term, but may have more detrimental effects in the long-run, when the balance between meaningful life and life itself is lost.

## References

Adams, V., Kaufman, S. R., van Hattum, T., & Moody, S. (2011). Aging Disaster: Mortality, Vulnerability, and Long-Term Recovery among Katrina Survivors. *Medical Anthropology*, *30*(3), 247–270. https://doi.org/10.1080/01459740.2011.560777

Albrecht, G. L., & Devlieger, P. J. (1999). The disability paradox: High quality of life against all odds. *Social Science & Medicine*, *48*, 977–988. https://doi.org/10.1016/S0277-9536(98)00411-0

Armitage, R., & Nellums, L. B. (2020). COVID-19 and the consequences of isolating the elderly. *The Lancet Public Health*, *5*(5), e256. https://doi.org/10.1016/S2468-2667(20)30061-X

Ayalon, L., Chasteen, A., Diehl, M., Levy, B. R., Neupert, S. D., Rothermund, K., Tesch-Römer, C., & Wahl, H.-W. (2020). Aging in Times of the COVID-19 Pandemic: Avoiding Ageism and Fostering Intergenerational Solidarity. *The Journals of Gerontology: Series B*, gbaa051. https://doi.org/10.1093/geronb/gbaa051

Backer, J. A., Mollema, L., Vos, R. A. E., Klinkenberg, D., van der Klis, F. R. M., de Melker, H. E., van den Hof, S., & Wallinga, J. (2020). *The impact of physical distancing measures against COVID-19 transmission on contacts and mixing patterns in the Netherlands: Repeated cross-sectional surveys in 2016/2017, April 2020 and June 2020* [Preprint]. Epidemiology. https://doi.org/10.1101/2020.05.18.20101501

Baltes, P. B., & Baltes, M. M. (1990). *Successful aging: Perspectives from the behavioral sciences*. https://doi.org/10.1017/CBO9780511665684

Barusch, A. S. (2011). Disaster, Vulnerability, and Older Adults: Toward a Social Work Response. *Journal of Gerontological Social Work*, *54*(4), 347–350. https://doi.org/10.1080/01634372.2011.582821

Becx, O. (2020, May 11). Tijdslot voor ouderen instellen [setting a curfew for older people]. *De Telegraaf*. https://www.telegraaf.nl/watuzegt/1896075824/tijdslot-voor-ouderen-instellen. Last accessed: 13 June 2020.

Bendien, E. & Abma, T. (2020). Silver lives matter. Available from: https://www.leydenacademy.nl/silver-lives-matter-how-intelligent-was-the-lockdown-for-dutch-older-people/. Last accessed: 10 October 2020.

Bernard, H. R. (2011). *Research methods in anthropology: Qualitative and quantitative approaches* (5th ed). AltaMira Press.

Blum, S., Brow, M., & Silver, R. C. (2012). Coping. In *Encyclopedia of Human Behavior* (pp. 596–601). Elsevier. https://doi.org/10.1016/B978-0-12-375000-6.00110-5

Bourdieu, P. (1986). The forms of capital. In *Handbook of Theory and Research for the Sociology of Education* (241-258). Greenwood.

Brooke, J., & Jackson, D. (2020). Older people and COVID-  19: Isolation, risk and ageism. *Journal of Clinical Nursing*, jocn.15274. https://doi.org/10.1111/jocn.15274

Bruinen de Bruin, Y., Lequarre, A.-S., McCourt, J., Clevestig, P., Pigazzani, F., Zare Jeddi, M., Colosio, C., & Goulart, M. (2020). Initial impacts of global risk mitigation measures taken during the combatting of the COVID-19 pandemic. *Safety Science*, *128*, 104773. https://doi.org/10.1016/j.ssci.2020.104773

Campbell, N. (2019). Disaster recovery among older adults: Exploring the intersection of vulnerability and resilience. In *Emerging Voices in Natural Hazards Research* (pp. 83–119). Elsevier. https://doi.org/10.1016/B978-0-12-815821-0.00011-4

Carver, C. (2013). Coping. In M. D. Gellman & J. R. Turner (Eds.), *Encyclopedia of Behavioral Medicine* (pp. 496–500). Springer New York. https://doi.org/10.1007/978-1-4419-1005-9_1635

Coleman, P. G., Ivani-Chalian, C., & Robinson, M. (1999). Self and Identity in Advanced Old Age: Validation of Theory Through Longitudinal Case Analysis. *Journal of Personality*, *67*(5), 819–849. https://doi.org/10.1111/1467-6494.00074

Coleman, P. G., McKiernan, F., Mills, M., & Speck, P. (2007). In sure and uncertain faith: Belief and coping with loss of spouse in later life. *Ageing and Society*, *27*(6), 869–890. https://doi.org/10.1017/S0144686X07006551

Donnellan, M. B., & Lucas, R. E. (2008). Age differences in the big five across the life span: Evidence from two national samples. *Psychology and Aging*, *23*(3), 558–566. https://doi.org/10.1037/a0012897

Fernandez, L. S., Byard, D., Lin, C.-C., Benson, S., & Barbera, J. A. (2002). Frail Elderly as Disaster Victims: Emergency Management Strategies. *Prehospital and Disaster Medicine*, *17*(2), 67–74. https://doi.org/10.1017/S1049023X00000200

Fry, P. S., & Keyes, C. L. M. (2010). Introduction. In C. L. M. Keyes & P. S. Fry (Eds.), *New Frontiers in Resilient Aging: Life-Strengths and Well-Being in Late Life* (pp. 1–14). Cambridge University Press; Cambridge Core. https://doi.org/10.1017/CBO9780511763151.002

Furedi, F. (2007). The changing meaning of disaster. *Area*, *39*(4), 482–489. https://doi.org/10.1111/j.1475-4762.2007.00764.x

Gabriel, Z., & Bowling, A. (2004). Quality of life from the perspectives of older people. *Ageing and Society*, *24*(5), 675–691. https://doi.org/10.1017/S0144686X03001582

Gerrard, N. (2020, October 5). How did we end up turning our care homes into jails of enforced loneliness? *The Guardian*. https://www.theguardian.com/commentisfree/2020/may/10/how-did-we-end-up-turning-our-care-homes-into-jails-of-enforced-loneliness

Glaser, BG. & Strauss, AL. (1967). *The Discovery of Grounded Theory: Strategies for Qualitative Research.* New York: Aldine De Gruyter. https://doi.org/10.1097/00006199-196807000-00014

Greve, W., & Staudinger, U. M. (2015). Resilience in Later Adulthood and Old Age: Resources and Potentials for Successful Aging. In D. Cicchetti & D. J. Cohen (Eds.), *Developmental Psychopathology* (pp. 796–840). John Wiley & Sons, Inc. https://doi.org/10.1002/9780470939406.ch21

Henderson, T. L., Roberto, K. A., & Yoshinori, K. (2010). Older adults responses to hurricane Katrina: Daily hassles and coping strategies. *Journal of Applied Gerontology*, *29*(1), 48-69. https://doi.org/10.1177/0733464809334287

Inouye, S. K., Studenski, S., Tinetti, M. E., & Kuchel, G. A. (2007). Geriatric Syndromes: Clinical, Research, and Policy Implications of a Core Geriatric Concept: (See Editorial Comments by Dr. William Hazzard on pp 794–796). *Journal of the American Geriatrics Society*, *55*(5), 780–791. https://doi.org/10.1111/j.1532-5415.2007.01156.x

Irvine, A. (2011). Duration, Dominance and Depth in Telephone and Face-to-Face Interviews: A Comparative Exploration. *International Journal of Qualitative Methods*, *10*(3), 202–220. https://doi.org/10.1177/160940691101000302

Irvine, A., Drew, P., & Sainsbury, R. (2013). 'Am I not answering your questions properly?' Clarification, adequacy and responsiveness in semi-structured telephone and face-to-face interviews. *Qualitative Research*, *13*(1), 87–106. https://doi.org/10.1177/1468794112439086

Folkman, S., & Moskowitz, J. T. (2004). Coping: Pitfalls and Promise. *Annual Review of Psychology*, *55*(1), 745–774. https://doi.org/10.1146/annurev.psych.55.090902.141456

Kirchher, J. & Charles, K. (2018). Enhancing the sample diversity of snowball samples: Recommendations from a research project on anti-dam movements in Southeast Asia. PLoS ONE 13(8): e201710. https://doi.org/10.1371/journal.pone.0201710

Klaiber, P., Wen, J. H., DeLongis, A., & Sin, N. L. (2020). The ups and downs of daily life during COVID-19: Age differences in affect, stress, and positive events. *The Journals of Gerontology: Series B*, gbaa096. https://doi.org/10.1093/geronb/gbaa096

Klerk, M. de, Plaisier, I., & Wagemans, F. (2020). *Welbevinden ten tijde van corona*. Sociaal en Cultureel Planbureau. https://www.scp.nl/publicaties/publicaties/2020/09/10/welbevinden-ten-tijde-van-corona.-eerste-bevindingen-op-basis-van-een-bevolkingsenquete-uit-juli-2020

Lazarus, R. S. (1993). From Psychological Stress to the Emotions: A History of Changing Outlooks. *Annual Review of Psychology*, *44*(1), 1–22. https://doi.org/10.1146/annurev.ps.44.020193.000245

Lichtenstein, R. (2020). From "Coffin Dodger" to "Boomer Remover": Outbreaks of ageism in three countries with divergent approaches to Coronavirus control. *The Journals of Gerontology: Series B, gbaa102,* https://doi.org/10.1093/geronb/gbaa102.

Liddell, J., & Ferreira, R. J. (2019). Predictors of Individual Resilience Characteristics Among Individuals Ages 65 and Older in Post-Disaster Settings. *Disaster Medicine and Public Health Preparedness*, *13*(02), 256–264. https://doi.org/10.1017/dmp.2018.52

Natale, F., Ghio, D., Tarchi, D., Goujon, A., & Conte, A. (2020). *COVID-19 Cases and Case Fatality Rate by age*. European Commission. https://ec.europa.eu/knowledge4policy/sites/know4pol/files/jrc120420_covid_risk_and_age.pdf

Neupert, S. D., Neubauer, A. B., Scott, S. B., Hyun, J., & Sliwinski, M. J. (2019). Back to the Future: Examining Age Differences in Processes Before Stressor Exposure. *The Journals of Gerontology: Series B*, *74*(1), 1–6. https://doi.org/10.1093/geronb/gby074

Nevedal, A. L., Ayalon, L., & Briller, S. H. (2019). A Qualitative Evidence Synthesis Review of Longitudinal Qualitative Research in Gerontology. *The Gerontologist*, *59*(6), e791–e801. https://doi.org/10.1093/geront/gny134

Ngo, E. B. (2001). When disasters and age collide: Reviewing vulnerability of the elderly. *Natural Hazards Review*, *2*(2), 80–89. https://doi.org/10.1061/(ASCE)1527-6988(2001)2:2(80)

Ngo, E. B. (2014). Elderly People and Disaster. In *Handbook of Hazards and Disaster Risk Reduction*. Routledge. https://doi.org/10.4324/9780203844236.ch37

Nugent, C. (2020, April 9). Governments are weighing how to ease Coronavirus lockdowns. Letting young adults out first could be one option. *Time*. https://time.com/5818593/young-people-leave-coronavirus-lockdown/

*Ontwikkeling COVID-19 in grafieken 22-12-2020 [Development of COVID-19 in charts 22-12-2020]*. (22-12-2020). RIVM. https://www.rivm.nl/coronavirus-covid-19/grafieken. Last accessed 22-12-2020.

Pinquart, M. (2002). Creating and maintaining purpose in life in old age: A meta-analysis. *Ageing International*, *27*(2), 90–114. https://doi.org/10.1007/s12126-002-1004-2

Powell, S., Plouffe, L., & Gorr, P. (2009). When ageing and disasters collide: Lessons from 16 international case studies. *Radiation Protection Dosimetry*, *134*(3–4), 202–206. https://doi.org/10.1093/rpd/ncp082

Richardson, J. C., Grime, J. C., & Ong, B. N. (2014). 'Keeping going': Chronic joint pain in older people who describe their health as good. *Ageing and Society*, *34*(8), 1380–1396. https://doi.org/10.1017/S0144686X13000226

Roberts, B. W., Walton, K. E., & Viechtbauer, W. (2006). Patterns of mean-level change in personality traits across the life course: A meta-analysis of longitudinal studies. *Psychological Bulletin*, *132*(1), 1–25. https://doi.org/10.1037/0033-2909.132.1.1

Ryff, C. D., & Singer, B. (1998). *The role of purpose in life and personal growth in positive human health.* Lawrence Erlbaum Associates Publishers.

Shaw, D., Scully, J., & Hart, T. (2014). The paradox of social resilience: How cognitive strategies and coping mechanisms attenuate and accentuate resilience. *Global Environmental Change*, *25*, 194–203. https://doi.org/10.1016/j.gloenvcha.2014.01.006

Skinner, E. A., Edge, K., Altman, J., & Sherwood, H. (2003). Searching for the structure of coping: A review and critique of category systems for classifying ways of coping. *Psychological Bulletin*, *129*(2), 216–269. https://doi.org/10.1037/0033-2909.129.2.216

Steptoe, A., Deaton, A., & Stone, A. A. (2014). Subjective wellbeing, health, and ageing. *The Lancet*. https://doi.org//10.1016/S0140-6763(13)61489-0.

van Gool, B. (2020, April 24). Lockdown kan ‘slim’ opgeheven worden voor iedereen jonger dan 50 toont onderzoek UvA aan [Lockdown can be relieved ‘smartly’ for everyone below age 50 shows research of UvA]. *EenVandaag*. https://eenvandaag.avrotros.nl/item/lockdown-kan-slim-opgeheven-worden-voor-iedereen-jonger-dan-50-toont-onderzoek-van-de-uva-aan/

Whitbourne, S. K. (2005). Successful Aging: Introductory Perspectives. *Research in Human Development*, *2*(3), 99–102. https://doi.org/10.1207/s15427617rhd0203_1

**Table 1. Participant characteristics**

| Characteristics | N=59[a] |
|---|---|
| **Demographic** | |
| Age (mean, range) | 75·5 (54 – 95) |
| Female | 34 (57·6%) |
| Married / widowed/ divorced/ living together/ single | 31 (52·5%)/ 13 / 4/ 3 / 8 |
| State pension only[b] | 7 (12·1%) |
| Migrant background | 17 (28·8%) |
| **Living situation** | |
| Living independent | 55 (93·2%) |
| Living in long-term care facility | 4 (6·9%) |
| Living alone/ with partner | 26 (44·1%)/ 33 |
| Living environment (city/ smaller city/ village) | 14 (23·7%)/ 30 / 15 |

[a] Data of 4 interviewees were excluded, 2 living in a long-term care facility and 2 with a migrant background, because of language and comprehension difficulties we were unable to ensure informed consent.

[b] National Old Age Pensions Act (*AOW*) is the Dutch state pension covering basic pension for everyone over age 67, 70% of the current minimum wage when living alone, 50% when living together if one lived in the Netherlands between the ages 15 and 65.
