# Supplementary material for: Coping of Older Adults in Times of COVID-19: Considerations of Temporality Among Dutch Older Adults
Source: J Gerontol B Psychol Sci Soc Sci. 2021 Jan 10;76(7):e290–9. doi: 10.1093/geronb/gbab008 (PMC7928596; doi:10.1093/geronb/gbab008)
Supplement: gbab008_suppl_Supplementary_Material [file gbab008_suppl_Supplementary_Material.docx]

**Supplementary material: Interview guide and topic list**

**Interview Guide**

The researcher asks a number of questions, qualitative research has an "open" character.

The researcher keeps an eye on the time, but is free to talk longer than the guideline indicates.

The goal is to answer the “why?” behind all individual questions. However, we prefer not to ask the why question literally, because this CAN cause rational and defensive answers.

**Introduction**

Start informally and reassure before proceeding to the "formal" meeting.

Explanation of the research:

Topics that will be discussed: your daily activities, relationships with others, keeping fit & healthy, and the news reporting about the virus and your personal view on the entire situation.

Request permission (vocally on tape) - privacy and anonymity

Collect background information: place of residence, age, living situation, partner.

Can you briefly introduce yourself?

**(Social) Activities**

I would like to start by talking with you about whether and how your daily life has changed since the corona measures/ restrictions were introduced.

Can you describe your daily life and the activities / hobbies that you took part in before corona?

What activities / things did you do? what activities did you participate in?

Outdoors / indoors?

What do these activities mean to you? What is important about this?

With whom did you do these activities?

Are there things [of the aforementioned activities / hobbies] that you are currently not able to do anymore due to the corona virus?

How do you feel about not being able to do this now?

Are there any activities that you are currently particularly missing? If so, can you tell us something about it. What do you miss?

Outdoors / indoors?

What would you need to be able to do this activity indoors? [online, via someone else]

Are there (new) activities / things that you have started to do since the corona measures were introduced?

Going deeper into the various activities; where / how / with whom does the activity take place?

What is the main motivation for you to do these activities / things?

Are there perhaps also activities or things that you are not doing yet but would like to do right now (in corona time)?

How would you like to do this?

**Social contacts and connectedness**

Much has changed because of the corona virus and measures. That is why I want to talk to you about the influence of the (new) measures on your (physical) contact with others.

How would you describe your contact with others before the corona virus and measures?

How did you maintain this contact? [physical visit, online, telephone]

Who were the most important contacts in your daily life?

How would you describe your contact with others now (since the corona measures)?

How do you feel about this? (what do you like about it? What do you like less about it?)

How do you maintain this contact? [physical visit, online, telephone]

Who are currently the most important people you have contact with in your (daily) life?

What topics do you currently have contact with others about? [stay connected, receive information, daily talk, etc.]

If not discussed: what influence do the measures have on your face-to-face contacts with others?

What significance does the contact that you have with others at this moment mean to you?

Who / what is important to you about the contact now?

Who / what do you find less important about the contact now

What are your main wishes regarding the contact with others at the moment?

Would you like to change anything? If so, who / what would you need to change this? What solutions could you think of in this way?

**Your well-being**

We are also curious about what concerns you as a person, what do you find important? What gives you pleasure in life?

Can you first tell something about yourself; how would you describe yourself as a person?

What kind of person would you like to be?

How has this changed since Corona?

Can you tell me what is important to you in your life?

Which things are important for you to have a happy / satisfied life? What make’s or would make you a happy / satisfied person?

When looking at your own life, how happy / satisfied are you now?

Has this changed since Corona?

**Vitality and health**

The corona measures can also affect your daily movement and physical health.

Can you tell what you are currently doing (i.e. since the corona measures) to stay physically healthy / fit?

How did you get into these activities? [tv, internet, own idea, etc.]

Where do you carry out these activities?

What do you need to carry out the activities?

How often do you do this on average per week?

What is the main motivation for you to stay fit?

If no physical activities: go to next question.

How do these [aforementioned daily physical activities] differ from your daily exercise / activities before the corona measures?

Does the amount of daily exercise match?

What would you need to get the same amount of daily exercise indoors?

Have you noticed a change in your physical health since the corona measures have taken effect?

What are your thoughts about your own health during the corona virus?

Positive or negative ideas / thoughts?

**Corona news & reporting**

Finally, I would like to talk a little bit about the corona virus media / government reporting and news.

What do you think of the local corona measures (briefly)?

How are you taking the measures yourself?

Risk to themselves or their loved ones?

Can you tell me how you follow the reports about the corona virus?

Media, social media, TV, news, etc.?

Can you tell me how you personally experience the news about the corona virus?

Continue on positive or negative terminology; examples? Certain media channels?

If we zoom in on the news about corona and the older population, how do you experience the reports / news?

What words come to mind when you think of this reports?

How do you think seniors are seen / portrayed in these messages?

Addressing the definition of older people as a "vulnerable target group"

**Final positive question:**

Finally, can you tell me the first thing that you will do once the corona measures/restrictions are lifted?

**Closing**

1. Do you have any questions or comments for me at the moment?

2. Repeating purpose, anonymity, etc.

3. Thank you very much for your participation and time.

4. Leave contact details if there is anything, additions

**Probs**

I don't quite understand that. Can you please explain that?

Can you please explain that? Explain further?

How does that work / how does that work exactly?

Can you tell more about it there / here?

Can you give an example?

Based on what experiences do you say that?

What do you mean exactly?

Can you describe what you mean?

With the probs it is important to consider the following:

- Expectations from family relationships are sometimes taken for granted and therefore not reported

- Social desirability; also because of shame culture, taboo, afraid of gossip

- Use of simple language

Silence: 5 seconds, take a moment to think about an answer

Repetition of the question.

If there really is no answer, why is this question so difficult? Can't answer?

Back to the question: thank you for sharing this, but I would still go back / to another aspect…

Thanks that is an interesting / useful addition. We have now discussed this aspect, I would like to…
